# Primary Conjunctival Melanoma of the Palpebral Conjunctiva: A Report of a Rare Case

**DOI:** 10.7759/cureus.117950

**Published:** 2026-10-15

**Authors:** Latifa Azarou, Othmane Zouiten, Leila Afani, Mohamed Elfadli, Rhizlane Belbaraka

**Affiliations:** 1 Oncology, Centre Hospitalier Universitaire (CHU) Mohammed VI, Marrakech, MAR; 2 Medical Oncology, Mohammed VI University Hospital of Marrakech, Marrakech, MAR; 3 Medical Oncology, Centre Hospitalier Universitaire (CHU) Mohammed VI, Marrakech, MAR

**Keywords:** conjunctival melanoma, melanocytic neoplasm, ocular tumor, palpebral conjunctiva, surgical excision

## Abstract

Conjunctival melanoma (CoM) is a rare but aggressive type of malignant ocular tumor with a wide spectrum of possible clinical presentations. Palpebral conjunctival localization is associated with special difficulties, as lesions may be hidden from visualization during ophthalmological examination, and therefore, the diagnosis may be delayed. Treatment should include a number of methods and consist of a complex approach, with surgical excision being the key point. According to tumor extension and histology, other treatment options, such as cryotherapy, topical chemotherapy, radiotherapy, immunotherapy, and targeted therapy, may be used as well. Prognosis is determined by tumor characteristics, local extension, and the completeness of surgical excision. We report a case of a patient with primary melanoma of the palpebral conjunctiva, which is a rather unusual presentation and has presented certain diagnostic and therapeutic challenges. A thorough clinical and imaging study of the lesion and subsequent surgical treatment and postoperative follow-up have been carried out. The described case demonstrates the need for a careful clinical examination of the conjunctiva, the assessment of the tumor extent, surgical excision, and multidisciplinary treatment. Follow-up should be long-lasting and meticulous to detect local recurrence and disease progression.

## Introduction

Conjunctival melanoma (CoM) is a rare form of malignant tumor, representing only 1%-2% of all ocular melanoma diagnoses worldwide. Malignant conjunctival melanoma arises from the abnormal proliferation of melanocyte cells in the conjunctiva (bulbar conjunctiva, caruncle, and palpebral conjunctiva) [1]. Although this type of tumor is relatively uncommon, the prognosis associated with malignancy remains poor and is significantly affected by the anatomic location of the CoM. The palpebral conjunctiva has the least favorable prognosis compared to other locations due to its complicated anatomic structure and difficulty when assessing the tumors via biomicroscopic examination. Furthermore, malignant tumors on the palpebral conjunctiva generally have a greater risk for recurrence compared to those occurring on the limbus or bulbus, requiring increased surveillance for recurrences. The mortality for patients with palpebral conjunctival tumors is greater than twice the mortality for patients with CoM found in other locations [2].

The majority of patients diagnosed with this condition are older adults, with the median age of diagnosis being around 62 years and a peak age range from 70 to 79 years. Like other melanoma types, ultraviolet (UV) exposure and light skin types are important environmental and genetic risk factors for the development of CoM. A patient case study is presented for a 59-year-old man with CoM of the left eye with palpebral conjunctival lesions, highlighting the unique diagnostic and treatment challenges associated with this location [3].

We present a case study of a 59-year-old man with palpebral conjunctival melanoma in the left eye to illustrate the diagnostic and therapeutic difficulties associated with this location, as well as management strategies that may aid treatment and prognosis.

## Case presentation

We report the case of a 59-year-old man with a medical history of well-controlled diabetes and occasional smoking, who presented in May 2025 to the Mohammed VI University Hospital Center in Marrakech, Morocco. The patient reported no significant history of sunlight exposure. He presented with ocular redness and persistent tearing for two months, without decreased visual acuity or other functional symptoms. Clinical examination revealed a round, pigmented, budding lesion of the upper eyelid tarsal conjunctiva. The patient had no known history of a pre-existing conjunctival nevus, primary acquired melanosis (PAM), or other ocular surface lesion at the site of the tumor. No precursor lesion was identified on clinical examination, and the lesion was therefore considered to have arisen de novo.

On ophthalmological examination, the patient presented with a round, pigmented, budding lesion on the tarsal conjunctiva of the upper left eyelid. The rest of the examination and all other ocular structures were normal. The bilateral fundus examination was normal; no posterior intraocular abnormalities were observed (Figure 1).

**Figure 1:**
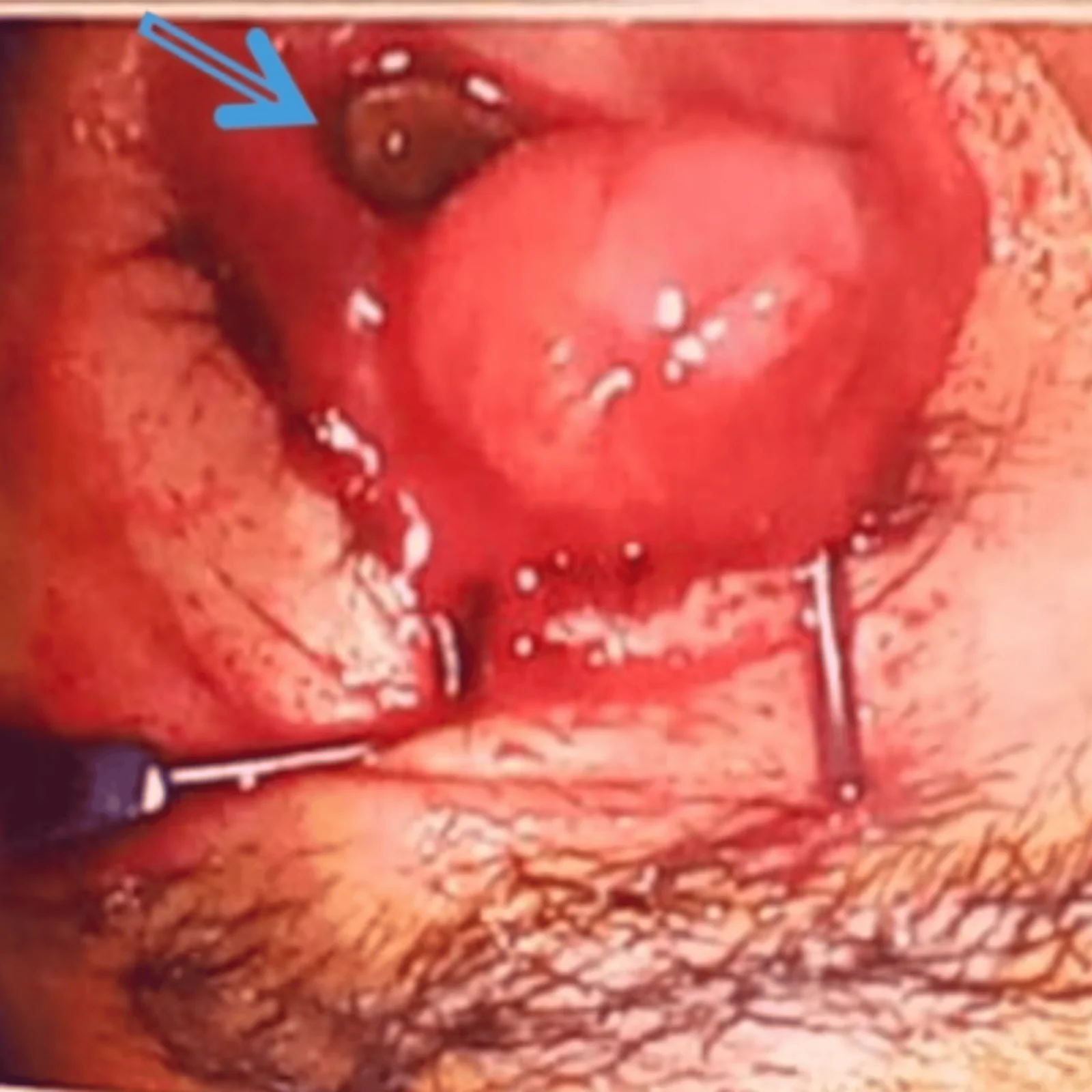
Intraoperative view showing a round, pigmented, budding lesion arising from the tarsal conjunctiva of the left upper eyelid (blue arrow).

A comprehensive clinical assessment was performed to exclude an extraocular primary melanoma. Dermatologic examination revealed no suspicious pigmented skin lesions, and the clinical examination did not identify another potential primary site.

Orbital MRI showed a well-defined nodular lesion of the superior left eyelid, measuring 12 × 8 mm. The mass was isointense on T1-weighted sequences and hyperintense on T2 fluid-attenuated inversion recovery (FLAIR), with diffusion restriction on apparent diffusion coefficient (ADC), and showed intense enhancement after contrast administration (Figure 2).

**Figure 2:**
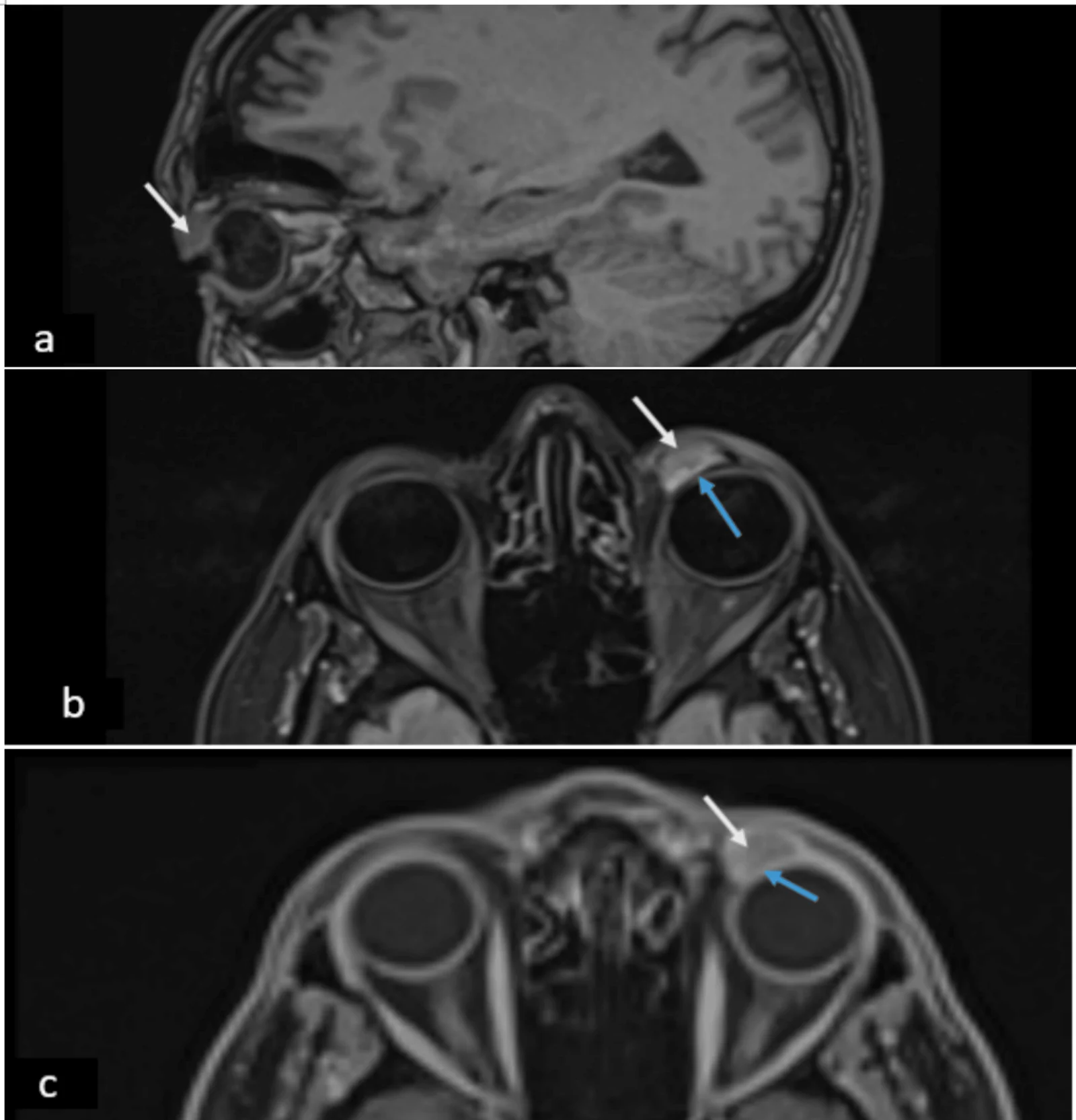
MRI: superior eyelid nodule of the left eye (white arrows), well-defined, showing isointense signal on T1 (a), hyperintense signal on T2 FLAIR (b), and enhancement after gadolinium injection (c), in contact posteriorly with the eyeball and broadly implanted on the sclera (blue arrows). FLAIR: fluid-attenuated inversion recovery

The lesion was removed by simple local excision, because the no-touch technique and adjunctive cryotherapy were not available at the time of surgery. The specimen arrived fragmented, so surgical margins could not be assessed.

Histopathological examination showed several fragments of tissue, aggregating to 1.4 g. The microscopic sections showed a malignant infiltrating tumor with superficial ulceration. Tumor cells were large and round, reflecting marked variation in cellular and nuclear size, with irregular chromatin and a spattering of nucleoli. Mitotic figures were plentiful. The cytoplasm was abundant and contained melanin pigment. The stroma consisted of tiny areas that were essentially fibrous, sprinkled with inflammatory cells. Surgical margins could not be evaluated due to fragmentation. Overall, these features were highly suggestive of an invasive and ulcerated conjunctival melanoma (Figure 3).

**Figure 3:**
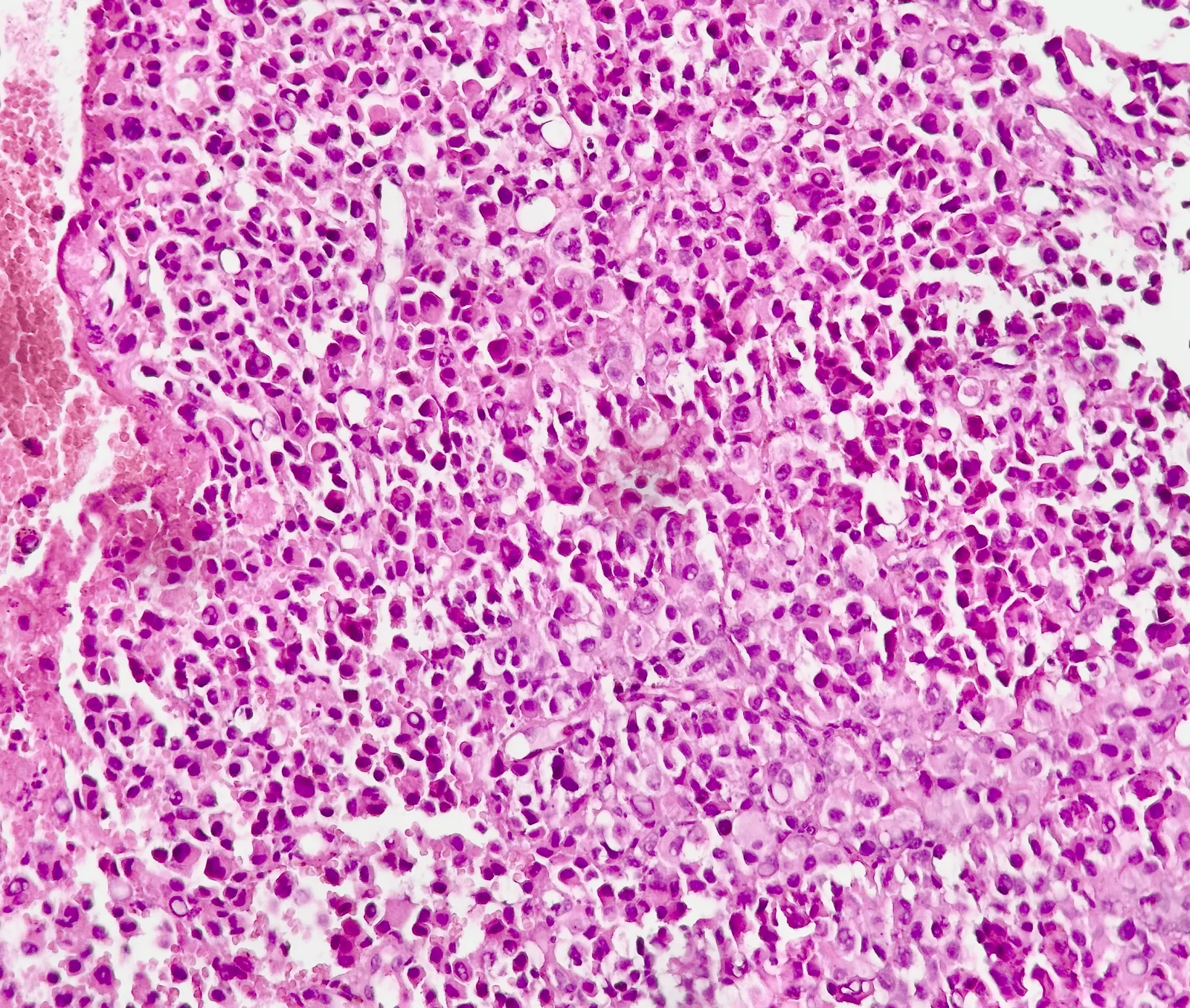
Tumor composed of large, pleomorphic, melanin-containing cells (H&E: ×100).

Immunohistochemical studies supported the diagnosis: tumor cells showed strong, diffuse positivity for S100; moderate positivity for HMB-45 (Figure 4); and a Ki-67 proliferation index of about 15%.

**Figure 4:**
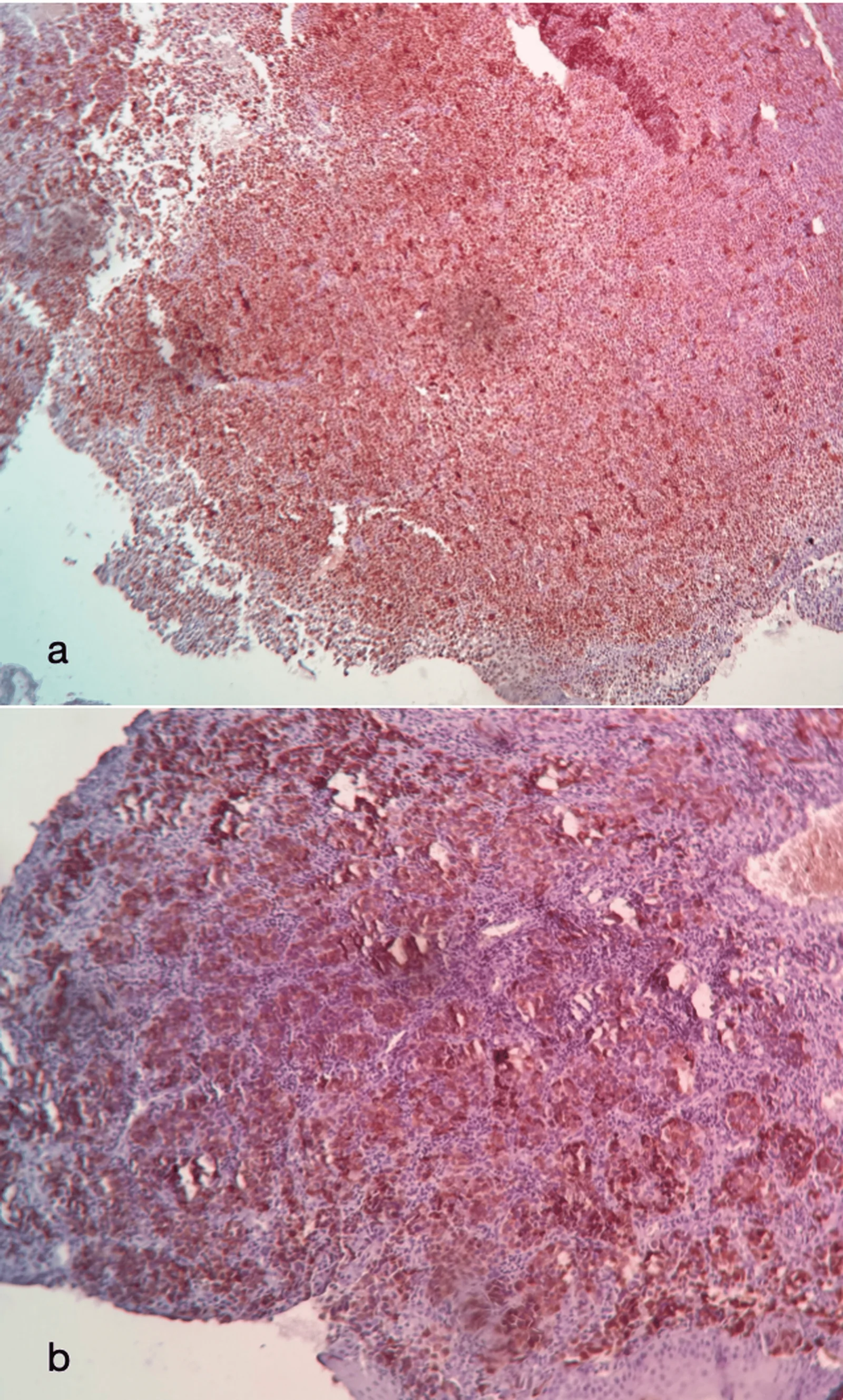
Microscopic features showing strong and diffuse positivity of tumor cells for PS100 antibody (a). Microscopic features showing positive nuclear staining of tumor cells by anti-HMB-45 antibody (b).

A diagnosis of invasive conjunctival melanoma was therefore established. A brain and orbit MRI performed postoperatively revealed no residual lesion (Figure 5).

**Figure 5:**
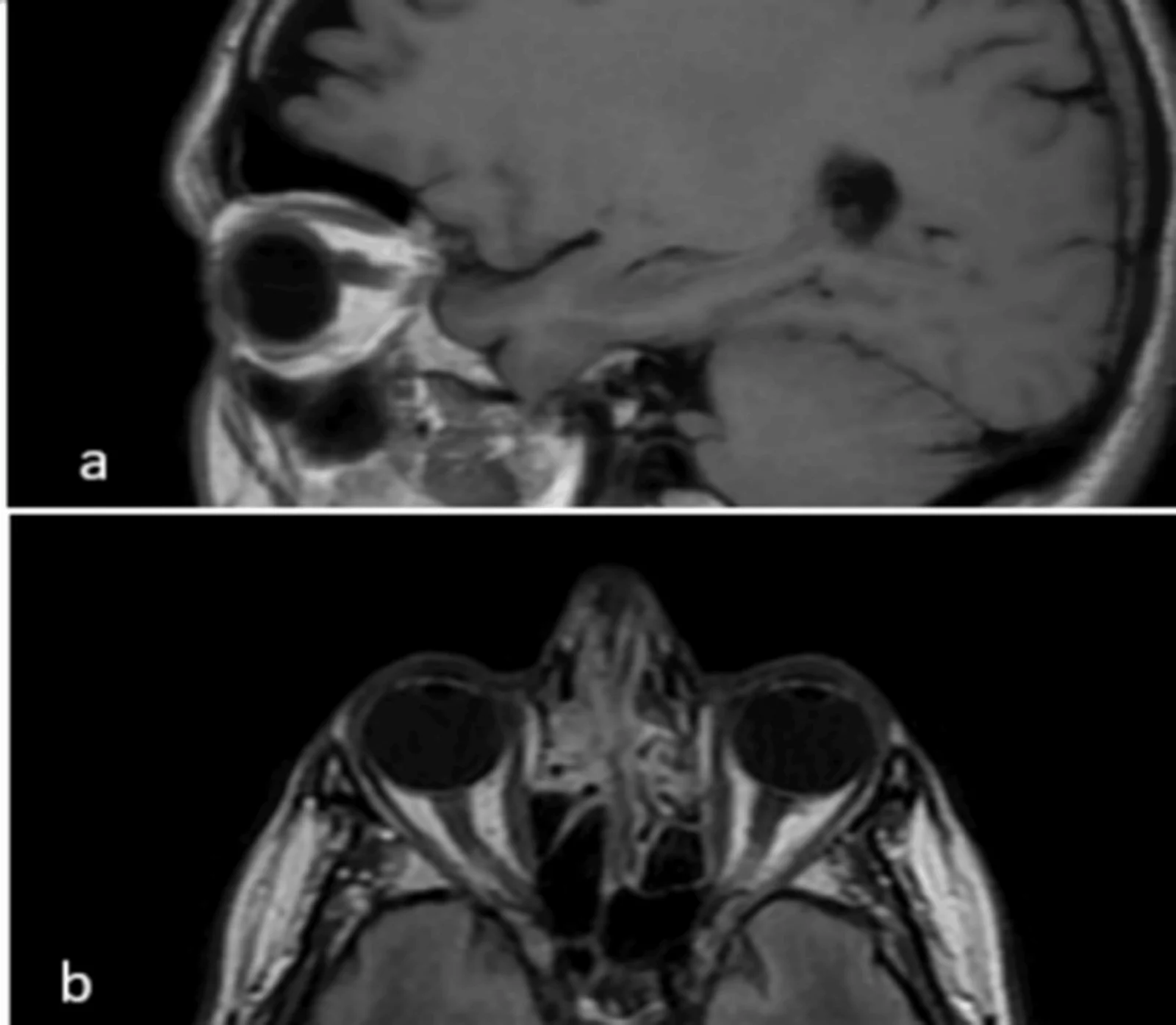
MRI: no nodular lesion on T1 (a) and T2 FLAIR (b). FLAIR: fluid-attenuated inversion recovery

For systemic staging, a thoraco-abdomino-pelvic computed tomography (CT) scan was performed and showed no evidence of distant metastatic disease.

Because the surgical specimen was fragmented, tumor thickness could not be reliably measured, and pathological T staging (pT) could not be assigned. Histopathological examination demonstrated an invasive and ulcerated melanoma. According to the American Joint Committee on Cancer (AJCC) eighth edition tumor-node-metastasis (TNM) classification for conjunctival melanoma, the tumor was clinically classified as cT2, as it arose from the non-bulbar (tarsal) conjunctiva of the upper eyelid. Preoperative orbital MRI showed a well-defined 12 × 8 mm nodular lesion with no evidence of invasion of the globe, eyelid, orbit, or adjacent structures, which excluded a higher T category. Regional lymph node status could not be assessed because neither sentinel lymph node biopsy nor regional lymph node dissection was performed (cNx). The absence of distant metastases on thoraco-abdomino-pelvic CT supported a clinical M0 classification (cM0).

After multidisciplinary discussion, surveillance alone was adopted because there was no residual lesion on postoperative MRI or on slit-lamp examination, adjuvant radiotherapy carried a high risk of ocular toxicity, and cryotherapy, brachytherapy, and topical chemotherapy were not available at our institution. Sentinel lymph node biopsy and PET-CT were not performed because they were not financially accessible to the patient. The patient consented to this strategy.

The patient has been followed for 15 months after surgical excision. During this period, regular complete ophthalmological examinations were performed, with no clinical evidence of local recurrence. Follow-up orbital MRI showed no evidence of local tumor recurrence. Thoraco-abdomino-pelvic CT performed during follow-up revealed no evidence of distant metastatic disease. At the most recent follow-up, 15 months after surgery, the patient remained clinically stable, with no evidence of local or distant disease. Continued long-term clinical and radiological surveillance is planned.

## Discussion

Malignant conjunctival melanoma (CoM) is an uncommon malignancy that derives from melanocytes. CoM accounts for approximately 2% of all ocular tumors, around 5% of melanomas arising in the ocular region, and roughly 0.25% of all melanomas. Although uncommon, CoM remains a notable cause of related mortality. Based on population data from national registries in Finland, Sweden, Denmark, the Netherlands, and the United States, the incidence of CoM is estimated to be between 0.24 and 0.8 cases per million individuals [4].

The majority of CoMs arise from pre-existing lesions, with approximately 75% developing from PAM+, 20% from conjunctival nevi, and 5% de novo. Ultraviolet (UV) exposure, fair skin, and increasing age are well-defined risk factors. Genetic alterations, including, but not limited to, mutations in TP53, BRAF, NRAS, or c-KIT, may also play a role, as does immunosuppression. Other, less clearly defined, risk factors include vitamin A deficiency, xeroderma pigmentosum, allergic conjunctivitis, tobacco use, and chemical exposures [1,5].

Clinically, CoM typically appears as a raised, nodular, pigmented lesion, either with feeding vessels or associated with areas of PAM. Diagnosis is made through clinical examination with a biomicroscope and confirmed by histopathology after an excisional biopsy, which is preferred. Incisional biopsy is reserved for larger lesions that cannot be excised [6]. Histologically, CoMs are seen to demonstrate atypical polyhedral, fusiform, or epithelioid melanocytes, with positive expression of Bcl-2, S100, MelanA, HMB-45, and Ki-67 to assess proliferative activity [7].

CoM preferentially metastasizes to regional lymph nodes in 45%-60% of cases [6]. PET-CT has been an important modality in evaluation and follow-up, with sensitivity of 81.3%, specificity of 98%, and accuracy of 94% [8]. Sentinel lymph node biopsy and PET-CT were not performed because they were not financially accessible to the patient.

Localized conjunctival melanoma is primarily treated by surgical excision with the no-touch technique, usually in combination with cryotherapy of the margins to minimize the risk of tumor dissemination and local recurrence. If the margins are positive or there is residual intraepithelial neoplasia, topical chemotherapy (mitomycin C or 5-fluorouracil) may be appropriate, and topical immunotherapy with interferon α2β could be used. Conjunctival melanoma is generally radioresistant, so radiotherapy is not used as primary treatment. As an adjuvant therapy after surgical excision, brachytherapy is delivered in multiple small fractions, typically using Sr⁹⁰ or I¹²⁵, at a mean total dose of ~47 Gy over 6-7 fractions (average 8 Gy per fraction) at a depth of 1.5-3 mm, with complete resolution in ~80% of cases. External beam radiation therapy (EBRT) or proton beam therapy is delivered at a mean total dose of ~51 Gy over ~9-10 fractions (average 4-5 Gy per fraction), with lower local control and higher toxicity. Focal cryotherapy is applied in a double freeze-thaw cycle at the surgical margins, particularly for recurrent or residual lesions, and it shows high rates of complete resolution with minimal risk of metastasis. Nevertheless, phase III trials of BRAF/MEK inhibitors and immune checkpoint inhibitors have shown improved relapse-free survival and reduced recurrence in cutaneous melanoma; however, conjunctival melanoma was excluded due to its rarity. Evidence from case reports and retrospective series indicates that BRAF-targeted therapy and PD-1 inhibitors can induce partial or complete responses in advanced conjunctival melanoma, with disease-free survival and overall prognosis apparently favorable in selected high-risk cases, although long-term outcomes remain uncertain [1,4,9]. In our patient, the initial procedure was a simple local excision rather than the recommended no-touch technique with adjunctive cryotherapy, because these were not available at the time of surgery. Specimen fragmentation therefore precluded margin assessment. Given the absence of residual disease on clinical and radiological follow-up, close surveillance was chosen after multidisciplinary discussion. Ocular exenteration was not indicated given the good local control. Brachytherapy could be considered but is not available in our country, and external radiotherapy carries a risk of ocular toxicity. This deviation and the resulting uncertainty about margin status and local recurrence risk represent a limitation of this report.

Palpebral melanoma is an aggressive tumor characterized by a high rate of recurrence, estimated between 36% and 45% at five years and between 31% and 59% at 10 years. Systemic metastases occur in 11%-16% of cases at five years and in 18%-26% at 10 years. The organs most frequently affected are the liver, lungs, brain, and skin, with occasional extension toward the orbit and the nasolacrimal system. Melanoma-related mortality averages 18%, whereas five-year survival rates range from 74% to 86% and go down to between 41% and 78% at 10 years [4]. Clinical predictors of poor prognosis include multifocal tumors, orbital invasion, recurrence, and the involvement of the caruncle, plica semilunaris, eyelid margins, and the conjunctiva of the eyelid or fornix. Histopathological predictors of poor prognosis include tumor thickness of >0.8 mm, the invasion of surgical margins, pagetoid spread, lymphovascular invasion, the presence of epithelioid cells, and a mitotic index of ≥5/10 high-power fields (HPF) [10,11].

## Conclusions

This case illustrates the challenges in the diagnosis and treatment of conjunctival melanoma, especially site-wise in an unusual area such as the tarsal conjunctiva. Early recognition and effective treatment are vital to improving prognosis. There is a need for more research to better understand the mechanisms of progression and refine treatment strategies, especially in the context of targeted therapies and immunotherapy. Improved access to local treatments would also help optimize local disease control in developing countries.
